# The Dimethyl Fumarate Experience: A Handy Drug With Broad Clinical Utility

**DOI:** 10.3389/fneur.2021.679355

**Published:** 2021-09-01

**Authors:** Lorena Lorefice, Elisa Casaglia, Marzia Fronza, Jessica Frau, Giuseppe Fenu, Silvy Pilotto, Giancarlo Coghe, Maria A. Barracciu, Eleonora Cocco

**Affiliations:** ^1^Department of Medical Sciences and Public Health, Multiple Sclerosis Center, Binaghi Hospital, ATS Sardegna, University of Cagliari, Cagliari, Italy; ^2^Radiology Unit, Binaghi Hospital, ATS Sardegna, Cagliari, Italy

**Keywords:** multiple sclerosis, dimethyl fumarate, real world study, efficacy, NEDA 3

## Abstract

**Objectives:** The aim of this study was to characterize multiple sclerosis (MS) patients exposed to dimethyl fumarate (DMF) and to evaluate the predictors of therapeutic response. In addition, the study offers a picture of how DMF use has changed over the past few years in naive or switcher patients.

**Methods:** In this observational monocentric study, we examined the prescription flow of DMF in MS patients categorized as naive or switchers (for safety/tolerability, ineffectiveness, and de-escalation strategy) from 2015 to 2019. Clinical and magnetic resonance imaging data of DMF-treated patients were analyzed, and NEDA-3 status at 24 months was evaluated by the three assessment components (absence of clinical relapses, no Expanded Disability Status Scale progression, no radiological activity). Determinants of therapeutic response were also evaluated using regression analysis.

**Results:** The sample included 595 MS patients exposed to DMF categorized as naive (158; 26.5%) and switchers for reasons of safety/tolerability (198; 33.3%), inefficacy (175; 29.4%), and de-escalation strategy (64; 10.8%). A 15% increase in DMF use in naive and horizontal shift groups was observed in the last 3 years of observation, whereas there was a drop, with prescription passed from ~20% to <5%, as an exit strategy from second-line therapies. NEDA-3 status was calculated for 340 patients after 24 months of DMF treatment and achieved in 188 (55.3%) of these. Analyzing the predictors of DMF response, we observed that lower annualized relapse rate (ARR) in 2 years pretreatment [hazard ratio (HR) = 0.49, *p* = 0.001] and being naive patients (HR = 1.38, *p* = 0.035) were associated with achievement of NEDA-3. Analogously, ARR in 2 years pretreatment affected the NEDA-3 achievement at 24 months in patients of the de-escalation group (HR = 0.07, *p* = 0.041), also indicating an effect related to the DMF initiation within 3 months (HR = 1.24, *p* = 0.029).

**Conclusion:** Our findings confirm DMF as a handy drug with broad clinical utility, with greater benefits for naive patients and horizontal switchers. Additionally, an increase in the flow of DMF prescriptions in these two groups of patients was also observed in our cohort.

## Introduction

In the last decade, many changes have marked the therapeutic scenario of multiple sclerosis (MS), with the introduction of new disease-modifying therapies with different mechanisms of action, efficacy, and safety profile, resulting in improved choice and steps toward a personalized therapy (1). Dimethyl fumarate (DMF) has been approved as a first-line oral agent for the treatment of relapsing MS, based on the phase III clinical trials data (2, 3). Since its entry into clinical practice setting, postmarketing studies and several real-world experiences have highlighted the multifaceted utility of DMF and added knowledge to identify the best candidate patients. Some studies have shown improved clinical and radiological outcomes, mostly in patients with moderate disease activity before treatment, with better effects in naive patients compared with switchers (4, 5). Moreover, a number of MS-related factors appear to be predictors of response to DMF treatment, such as a shorter disease duration that has been associated with higher rate of NEDA-3 (No Evidence of Disease Activity) (6, 7). This point is in line with the assumption about the influence of the disease-modifying therapies on MS that indicates that “treating early is better than late, but late is better than never,” and this is fundamental to define the best choice and window of therapeutic opportunity (8). With the growing experience in the clinical setting, the use of DMF has changed, and the drug is increasingly considered as an option in naive patients and switchers, also in consideration of the data of comparative studies (9, 10), and it has also been evaluated as a possible exit strategy from second-line therapies (11, 12).

Based on these considerations, the present study aimed to (i) define demographic and clinical features of MS patients undergoing DMF therapy categorized as naive or switchers (for safety/tolerability, ineffectiveness, de-escalation strategy), also describing how DMF prescription flow has changed in these four patient categories over the past 5 years, and (ii) evaluate the efficacy data in the different DMF patient groups, also evaluating the predictors of therapeutic response.

## Methods

### Study Design and Data Acquisition

This is an observational monocentric study that included MS patients diagnosed with the revised McDonald criteria (13), who started DMF therapy between January 2015 and December 2019. The patients' demographic characteristics (sex and age) and clinical data [age at DMF initiation, disease duration, and disability level, evaluated using the Expanded Disability Status Scale (EDSS)] (14) were collected. The last follow-up of the year 2020 was considered for each patient. Previous disease-modifying therapies, date of last therapy withdrawal and reason of switching to DMF as well as the number of relapses, and annualized relapse rate (ARR) 2 years before DMF start were also recorded. Thus, patients were classified as naive or switchers due to three different reasons (safety/tolerability, ineffectiveness, de-escalation strategy). Additional information about the duration of DMF treatment, the number of relapses, the ARR during the DMF exposure, and magnetic resonance imaging (MRI) outcomes, such as presence of new or enlarging T2 lesions or gadolinium-enhancing T1 lesions at MRI assessments carried out annually after DMF initiation and compared with the rebaseline MRI performed after 6 months, were recorded. The timing of the rebaseline MRI was defined at 6 months, on the pharmacodynamics of the DMF, as recommended, to avoid considering disease activity that may occur in the weeks and months following the initiation of therapy as disease activity unresponsive to treatment. Next, for patients exposed to DMF for 24 months, NEDA-3 status was evaluated by the three assessment components (absence of clinical relapses, no EDSS progression, absence of radiological activity on MRI performed at 24 months of DMF compared to the rebaseline MRI), and determinants of NEDA-3 status were explored (6). Finally, all side effects reported by the patients were registered, as well as the DMF discontinuation causes and subsequent therapeutic choices. Informed consent was obtained from all participants after the local ethics committee approval.

### Statistical Analysis

SPSS for Mac version 20.0 (SPSS Inc., Chicago, IL, USA) was used to perform the statistical analysis. Descriptive statistics are presented using mean, SDs, and frequencies (absolute and relative). First, the percentage of naive patients and switchers initiated to DMF therapy was assessed for the years 2015–2019. Thereafter, demographic (sex, age) and clinical differences (disease duration, EDSS score, age at DMF initiation, and DMF duration) among patients exposed to DMF categorized as naive or switchers (for safety/tolerability; ineffectiveness; de-escalation strategy) were evaluated using independent-samples *t*-tests for quantitative variables and χ^2^-tests for qualitative variables. Mann–Whitney *U* tests were used to compare the ARR calculated 2 years before DMF therapy and at 24 months following DMF therapy for the four groups of patients, naive and switchers, also categorized in relation to the last disease-modifying therapy. Therefore, the achievement of NEDA-3 status at 24 months was calculated as a percentage of patients with no clinical relapses, EDSS progression, and radiological activity, and the predictors of NEDA-3 status were investigated using binary regression analysis. For all assays, statistical significance was set at *p* < 0.05.

## Results

The sample included 595 MS patients exposed to DMF categorized as naive (158; 26.5%) and switchers for reasons of safety/tolerability (198; 33.3%), inefficacy (175; 29.4%), and de-escalation strategy (64; 10.8%). Of the patient group, the mean DMF exposure was 28.7 (SD = ±18) months, while the median was 27 months (±32 months of IQR).

**Figure 1:**
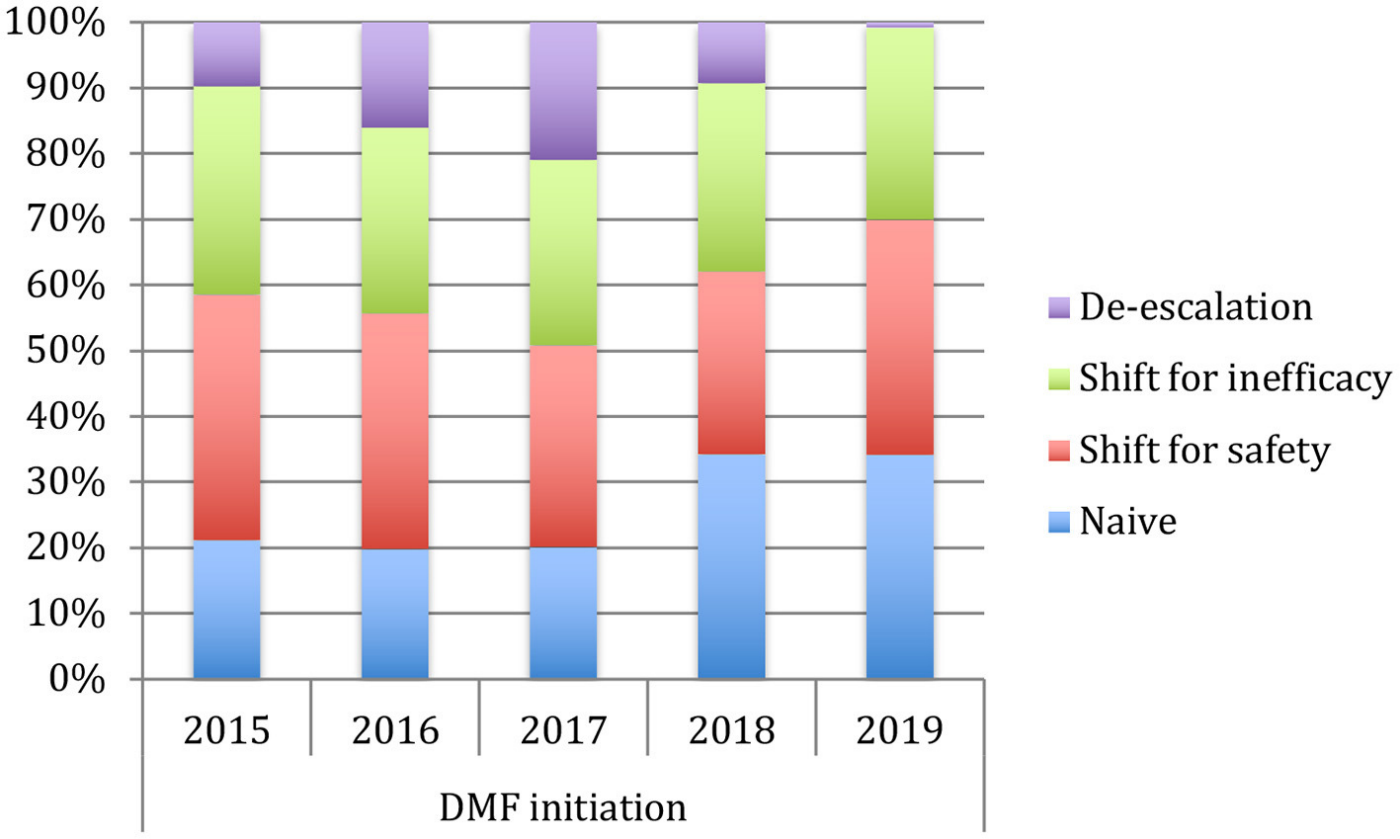
Use of dymetilfumarate in the last five years (2015–2019). The graph indicates the percentage of patients naive or who have undergone a therapeutic shift from 2015 to 2019.

Table 1 shows the demographic and clinical differences of naive MS patients versus switchers examined by χ^2^ and independent-samples *t*-tests, showing lower age, MS duration, and EDSS score (*p* < 0.005) for naive patients. The percentage of naive MS patients who initiated DMF use is detailed in Figure 1; in particular, in the last 2 years of the observation period (2015–2019), there was a 15% increase in the use of DMF in naive subjects, prescribed in ~20% of patients initiated on DMF between 2015 and 2017 and then in ~35% during 2018–2019. Similarly, an increase in DMF use in horizontal shift was observed in the last 3 years of observation, whereas there was a significant drop in DMF use as an exit strategy, with prescription in ~20% of patients during 2017, in ~10% during 2018, and then in <5% during 2019. MS treatments before DMF initiation are detailed in Table 2. In particular, among the 437 switchers patients, a shift for safety/tolerability was reported by 198 patients [144 (72.7%) after interferon β, 35 (17.7%) after glatiramer acetate, 19 (9.6%) after teriflunomide], whereas a shift for inefficacy was reported by 175 subjects [111 (63.4%) after interferon β, 46 (26.3%) after glatiramer acetate, 18 (10.3%) after teriflunomide]. DMF as exit strategy from second-line therapies was used by 64 patients; of these, 56 (87.5%) shifted from natalizumab for JC virus antibody seropositivity and 8 (12.5%) from fingolimod, with mean time from second-line treatment to DMF initiation of 121 ± 87 days. Of de-escalating patients, four had a relapse in the wash out period, while five within the first year.

**Table 1:** Demographic and clinical features of MS patients exposed to dimethylfumarate categorized as naive or based on the type of therapeutic shift (horizontal for safety; horizontal for DMDs ineffectiveness; de-escalation).

	**MS Patients exposed to Dymetilfumarate (595)**				
	**Naïve (158; 26.5%)**	**Switchers (437; 73.5%)**	**Horizontal shift for safety (198; 33.3%)**	**Horizontal shiftfor DMDs inefficacy (175; 29.4%)**	**De-escalation Shift (64; 10.8%)**
Male Gender	47 (29.7%)^*^	112 (70.9%)	46 (23.2%)	50 (28.1%)	16 (25%)
Age at DMF initiation (years)	35.9 ± 10.6^**^	40.3 ± 9.8	40.6 ± 9.3	39.9 ± 10.7	41.2 ± 8.5
MS duration at DMF initiation (years)§	2.9 ± 4.7^**^	10.3 ± 7.5	10.1± 7.2	9.3 ± 7.5	13.9 ± 7.8
EDSS score at DMF initiation	1.7 ± 1.1^**^	2.4 ± 1.6	2.2± 1.5	2.2 ± 1.5	3.3 ± 2.1
DMF exposition (months)	25.9 ± 18.3^*^	29.8 ± 17.7	29.2 ± 18.3	30.1 ± 18.2	30.8 ± 14.5

* *p-value 0.05*.

** *p <0.005*.

*§MS duration at DMF initiation (years) is defined with respect to the first MS symptom presentation*.

*Chi-square and independent-samples t-tests were used to compare demographic and clinical features of naïve MS patients vs. MS patients previously treated with DMDs (switchers). Data for each group of switchers are also shown*.

**Table 2:** DMDs treatment before dymetilfumarate initiation in relation to the type of therapeutic shift performed.

	**MS Patients exposed to therapeutic shift (437)**	
	**Horizontal shift for safety (198)**	**Horizontal shift for DMDs inefficacy (175)**
INF **β**	144 (72.7%)	111 (63.4%)
Glatiramer Acetate	35 (17.7%)	46 (26.3%)
Teriflunomide	19 (9.6%)	18 (10.3%)
	**De-escalation shift (64)**	
Fingolimod	8 (12.5%)	
Natalizumab	56 (87.5%)	

Table 3 shows the comparisons between ARR 24 months before and after DMF therapy, analyzed for 340 MS patients (naive or switchers), indicating a significant ARR reduction in the naive group (ARR pre-DMF 0.30 ± 0.34 vs. ARR post-DMF 0.19 ± 0.36, *p* = 0.014), switchers for inefficacy (ARR pre-DMF 0.67 ± 0.68 vs. ARR post-DMF 0.11 ± 0.18, *p* = 0.001), and switchers for safety/tolerability (ARR pre-DMF 0.47 ± 0.65 vs. ARR post-DMF 0.12 ± 0.24, *p* = 0.001). No difference in ARR before and after 24 months of DMF therapy was found in the de-escalation group, which continued DMF treatment. However, nine de-escalating patients (five after natalizumab and three after fingolimod) discontinued DMF within the first year, whereas 10 patients (six after natalizumab and four after fingolimod) discontinued DMF between the first and second year, mainly due to ineffectiveness (72.2%).

**Table 3:** ARR before and after 24 months of DMF (340MS patients).

	**ARR 2 years pre DMF**	**ARR on DMF**	***p*-value**
**MS patients–Naive (77)**			
	**0.30** **±** **0.34**	**0.19** **±** **0.36**	**0.014**
**MS patients–Horizontal shift for safety (114)**			
IFN (82)	0.47 ± 0.70	0.12 ± 0.25	<0.001
GA (17)	0.47 ± 0.59	0.08 ± 0.15	0.043
TFU (15)	0.50 ± 0.52	0.15 ± 0.26	0.057
TOT	**0.47** **±** **0.65**	**0.12** **±** **0.24**	** <0.001**
**MS patients–Horizontal shift for DMDs inefficacy (106)**			
IFN (71)	0.67 ± 0.66	0.10 ± 0.17	<0.001
GA (23)	0.52 ± 0.39	0.15 ± 0.22	<0.001
TFU (12)	1.0 ± 1.15	0.08 ± 0.17	0.001
TOT	**0.67** **±** **0.68**	**0.11** **±** **0.18**	** <0.001**
**MS patients–De-escalation shift (46)**			
NTZ (42)	0.13 ± 0.33	0.19 ± 0.26	ns
FTY (4)	0.25 ± 0.35	0.26 ± 0.37	ns
TOT	**0.17** **±** **0.32**	**0.13** **±** **0.26**	**ns**

*Mann–Whitney tests were used to compare the ARR calculated 2 years before DMF and the ARR at 24 months of DMF for the four groups of patients (naive and switching to DMF)*.

*The significant results were shown in bold*.

**Figure 2:**
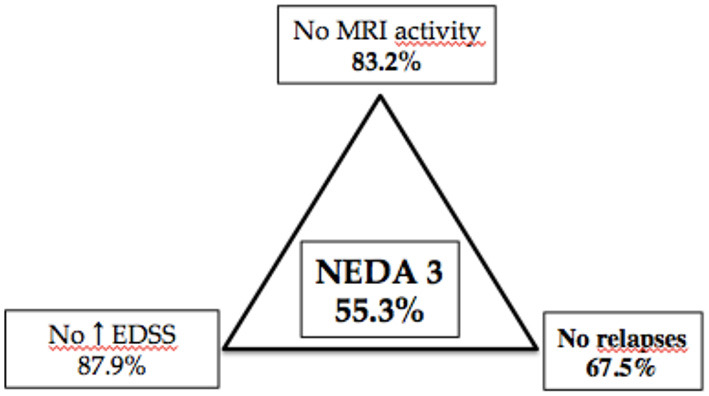
Different components of NEDA 3 status at 24-month follow-up in 340 patients with MS treated with dymethilfumarate.

Finally, NEDA-3 status was calculated for 340 patients after 24 months of DMF treatment and achieved in 188 (55.3%) of these. In detail, relapse-free status was observed in 229 patients (67.5%), no disability progression in 299 (87.9%), and MRI NEDA-3 status in 283 patients (83.2%) (Figure 2). Analyzing the predictors of response to DMF, we observed that lower ARR in the 2 years pretreatment [hazard ratio (HR) 0.49, *p* = 0.001)] and being naive patients (HR = 1.38, *p* = 0.035) were associated with achievement of NEDA-3 (Table 4). Analogously, ARR in the 2 years pretreatment affected the NEDA-3 achievement at 24 months in the patients of de-escalation group (HR = 0.07, *p* = 0.041), also indicating an effect related to the DMF initiation within 3 months (HR = 1.24, *p* = 0.029; Table 5).

**Table 4:** NEDA 3. Predictors of therapeutic response in patients exposed to 24 months of dymetilfumarate.

			**NEDA 3**			
				**95% C.I. for EXP (B)**		
		**B**	**Exp (B)**	**Lower**	**Upper**	***p***
Variables	Age at DMF initiation	0.008	1.010	0.982	1.035	0.542
	ARR 2 year pre DMF	−0.588	0.491	0.415	0.744	**0.001**
	MRI activity 2yr pre DMF	0.562	1.121	0.947	3.246	0.074
	Naive	0.662	1.389	0.275	0.967	**0.035**

*Multiple regression analysis was used to examine which demographic and clinical variables, included in the model as independent variables, influence the achievement of NEDA 3 status at 24 month of DMF exposure*.

*The significant results were shown in bold*.

**Table 5:** NEDA 3. Predictors of DMF efficacy after de-escalation switching.

			**NEDA 3**			
				**95% C.I. for EXP (B)**		
		**B**	**Exp (B)**	**Lower**	**Upper**	***p***
	ARR 2 year pre DMF	−2.225	0.070	0.13	0.915	0.041
	MRI activity 2 year pre DMF	−1.092	0.257	0.063	1.776	0.335
	DMF start within 3 months	1.244	1.151	1.139	10.571	0.029

*Regression analysis was used to examine clinical variables, included in the model as independent variables, influence the achievement of NEDA 3 status in MS patients switching from Natalizumab*.

The overall discontinuation rate was of 17.9% (107/595 patients); of these, 60 of 595 patients (10%) discontinued DMF due to ineffectiveness, 34 (5.7%) of whom within the first year of treatment [nine de-escalating from second-line Disease Modifying Treatments (DMTs)]. Analogously, of 21 patients (3.5%) who discontinued DMF between the first and second year, 10 were after de-escalation strategy.

Finally, 47 of 595 patients (7.9%) in our cohort discontinued DMF for safety/tolerability reasons, mainly during the first year of treatment (72% of cases). Of these, gastrointestinal (GI) symptoms were reported as the primary side effect, accounting for 4.6% of drug suspension, followed by flushing (3.1%) and laboratory testing abnormalities (hypertransaminasemia for 0.1% and prolonged lymphopenia for 0.1%). A shift to oral teriflunomide was reported in 18 (38.3%) of these patients, to glatiramer acetate (Copaxone) in 15 (31.9%), and to interferon in 11 (23.4%), whereas 3 patients (6.4%) did not undertake other immunotherapies.

## Discussion

Previous studies have evaluated the effectiveness of DMF with analysis of the real-world data. Our data can be differentiated from previous studies in several respects, including data source, cohort (this is a large real-world monocentric study), method of analysis (differentiated assessment of clinical outcomes for patients categorized into four groups), and the evaluation of the results with the examination of predictors of MS outcomes.

In line with other studies that have shown a good efficacy profile of DMF both in naive and horizontal switchers (4, 5, 7), we report a reduction of ARR in these two patient groups after 24 months of treatment. In keeping with this point, an increase in the prescription flow of DMF in naive and switchers for ineffectiveness or safety was observed during the observational period in our cohort, based on DMF persuasive efficacy–risk profile as well as patient preference for oral administration. Furthermore, our data offer new evidence in clinical setting on horizontal therapeutic switching choice, on which a growing literature is trying to discuss the utility of the use of drugs of the same line with different mechanisms of action, as well as the best time and patient candidates for this choice (15, 16). On the contrary, a reduction in DMF use as exit strategy from second-line therapies was reported in our cohort. Bearing in mind that patients de-escalating from second-line therapies to DMF did so mostly for safety reasons, in particular for JC virus positivity during natalizumab treatment, in line with published data (11, 12, 17), we observed that DMF did not eliminate the risk of MS reactivation, with discontinuation of DMF during the first year for 8.9% of our patients previously treated with natalizumab. Interestingly, for patients who persist in DMF treatment, no differences in pre- and post-ARR at 24 months were observed. Moreover, the regression analysis showed that the latency in months in the initiation of DMF therapy is an important determinant of the achievement of NEDA-3 at 24 months, reinforcing the concept of the need to rapidly finalize the therapeutic choice (18), in particular in de-escalation switching.

The reduction of DMF use as an exit strategy observed in our cohort may be attributable to a better selection of patients to be initiated on natalizumab therapy based on JC virus serostatus (19), as well as to the use of new strategies in the clinical setting that allow to continue the treatment while limiting the risk of progressive multifocal leukoencephalopathy (i.e., natalizumab extending dose protocol) (20, 21). However, it is conceivable that the reduction of DMF use as de-escalating strategy is also attributable to the recent availability of more efficacious agents with rapid effects (21), as well as to the growing awareness that the timing of full effectiveness of DMF does not prevent from disease rebound (11).

Analyzing the predictors of NEDA-3 status, clinical activity in the 2 years preceding DMF and being naive patients emerged as significant determinants confirming, as previously demonstrated by Lanzillo et al., the utility of this oral agent from the earliest stages of the disease (7). Analogously, other studies showed that not only naive patients strongly benefit from DMF, but also patients switched from injectable DMTs due to tolerability and efficacy issues (4, 5). Moreover, our results showed a higher NEDA3 proportion than that reported in the Northern Italy Multicenter Study (5), and this likely is attributable to the differences in patient characteristics and selection. Similarly, we found a higher NEDA3 status of those described in the integrated analysis of the phase III DEFINE and CONFIRM studies (22). Finally, other studies of real-world setting found a higher baseline EDSS, a larger number of T1Gd+ lesions, and a switch because of inefficacy (vs. adverse events) as the principal risk factors for losing NEDA-3 status (23).

Overall, safety data confirmed a favorable profile for DMF, with 7.9% patients dropped out due to safety or tolerability issues, the most frequent being GI tolerability (4.6%). Grade III lymphopenia, which other studies reported as an infrequent event ranging between 3 and 15% of patients (4), was a rare cause of DMF discontinuation in our study (0.01%). Moreover, few recent studies explored the recovery of lymphocyte count after DMF discontinuation that could be very slow in some cases with potential consequences on treatment choice (24, 25). The evaluation of these and other safety aspects is of central importance to better understand adherence, treatment persistence, and the usefulness of other therapeutic decisions.

Another important safety issue linked to the increasingly widespread use of DMF in young women is related to pregnancy, on which preliminary data would have shown safe outcomes (26). Further data, to support these early evidences, are, however, needed.

The present study has several limitations mainly due to its retrospective nature. However, compared to other studies on this topic, the monocentric nature of our study allowed limiting the variability in radiological and clinical data collection. Furthermore, the study focused exclusively on evaluating DMF efficacy outcomes, considering the composite evaluation of clinical relapses, EDSS progression, and neuroradiological activity (NEDA-3) as disease outcome. Safety aspects of DMF and the possible predictors of safety outcomes have not been deliberately explored.

## Conclusion

Our findings confirm DMF as a handy drug with broad clinical utility. DMF use has progressively increased in clinical practice, showing greater benefits for naive patients and horizontal switchers. Further studies are needed to better investigate the predictors of efficacy, as well as the predictive biomarkers, for the best identification of patients to be initiated on DMF treatment, in the modern perspective of an effective, early, and personalized therapy (1).

## Data Availability Statement

The raw data supporting the conclusions of this article will be made available by the authors, without undue reservation.

## Ethics Statement

The studies involving human participants were reviewed and approved by University of Cagliari. The patients/participants provided their written informed consent to participate in this study.

## Author Contributions

LL and ECo conceptualized the study and wrote, reviewed, and edited the manuscript. ECa, MF, JF, GF, GC, SP, and MB were responsible for resources and data curation. All authors contributed to the article and approved the submitted version.

## Conflict of Interest

LL, JF, GF, GC, and ECo received honoraria for consultancy or speaking from Biogen, Novartis, Sanofi, Genzyme, Serono and Teva, and Almirall. The remaining authors declare that the research was conducted in the absence of any commercial or financial relationships that could be construed as a potential conflict of interest.

## Publisher's Note

All claims expressed in this article are solely those of the authors and do not necessarily represent those of their affiliated organizations, or those of the publisher, the editors and the reviewers. Any product that may be evaluated in this article, or claim that may be made by its manufacturer, is not guaranteed or endorsed by the publisher.
